# The Persuasive Power of Knowledge: Testing the Confidence Heuristic

**DOI:** 10.1037/xge0000471

**Published:** 2018-08-23

**Authors:** Briony D. Pulford, Andrew M. Colman, Eike K. Buabang, Eva M. Krockow

**Affiliations:** 1 Department of Neuroscience, Psychology and Behaviour, University of Leicester; 2 Institute of Psychology, Leiden University; 3 Department of Neuroscience, Psychology and Behaviour, University of Leicester

**Keywords:** computer-mediated communication, confidence heuristic, coordination, dominance, persuasion

## Abstract

According to the confidence heuristic, people are confident when they know they are right, and their confidence makes them persuasive. Previous experiments have investigated the confidence–persuasiveness aspect of the heuristic but not the integrated knowledge–confidence–persuasiveness hypothesis. We report 3 experiments to test the heuristic using incentivized interactive decisions with financial outcomes in which pairs of participants with common interests attempted to identify target stimuli after conferring, only 1 pair member having strong information about the target. Experiment 1, through the use of a facial identification task, confirmed the confidence heuristic. Experiment 2, through the use of geometric shapes as stimuli, elicited a much larger confidence heuristic effect. Experiment 3 found similar confidence heuristic effects through both face-to-face and computer-mediated communication channels, suggesting that verbal rather than nonverbal communication drives the heuristic. Suggesting an answer first was typical of pair members with strong evidence and might therefore be a dominant cue that persuades. Our results establish the confidence heuristic with dissimilar classes of stimuli and through different communication channels.

Consider a familiar scenario in which two friends, Ava and Ben, are deciding which of two possible restaurants to visit for a meal: Thai Break or Pasta Milestone. Thai Break is cheaper and has food that both would enjoy more than Pasta Milestone. Ben has no relevant knowledge on which to base a preference, whereas Ava has heard reports from her sister, who has eaten at both restaurants and found Thai Break to be much better. It is reasonable to suppose that the outcome would be best for both if they went to Thai Break, second-best for both if they went to Pasta Milestone, and worst for both if they failed to agree and consequently missed out entirely on a meal together. While discussing the options, it is likely that Ava would express a preference with greater confidence than would Ben; and even if she didn’t mention why she preferred Thai Break, it would be in Ben’s interest to interpret her confidence as evidence of superior knowledge and to defer to it. Ava and Ben would therefore probably end up dining at Thai Break.

According to the confidence heuristic, when people communicate beliefs to one another, they generally express confidence in proportion to their degree of certainty on the basis of their relevant knowledge; and if they have common interests in coordinating their decisions, recipients tend to judge the persuasiveness of the communication according to the confidence with which it is expressed. Thomas and McFadyen (1995), who introduced the heuristic as a purely theoretical proposal, also showed mathematically that the confidence heuristic permits efficient exchange of information between decision makers with common interests and that it reliably implements optimal solutions to interactive decisions characterized by shared preferences: technically, *common-interest games* in which one outcome Pareto dominates all other possible outcomes, and asymmetric information. The players’ interests coincide, and they are motivated to coordinate their actions, but they have incomplete information of varying reliability about the payoffs associated with the possible outcomes. Examples include life partners deciding between different houses to buy or politicians choosing between political leaders, where agreement is preferable to disagreement, and some jointly agreed on alternatives are typically preferable to others, but the individuals involved have different information about the available alternatives. The heuristic applies to common-interest games with asymmetric information, not to interactions in general. For example, a used-car dealer may tell a potential customer confidently that “this car is good for at least five more years,” but the customer is unlikely to be persuaded by the confidence of the communication because, although the dealer may know more than the customer, the two individuals obviously have diverging, rather than common, interests.

The formal properties of the confidence heuristic are outlined in the Appendix. The heuristic takes the form of a simple game-theoretic model of interactive decision making, but it is intended to be applicable to relevant joint decisions studied in social psychology. It rests on an assumption that people sometimes base their judgments of the persuasiveness or reliability of information communicated to them by others on the confidence with which it is expressed, judging information to be more persuasive if it is communicated confidently rather than tentatively. A second assumption introduced by Thomas and McFadyen (1995) is that there exists a social norm according to which people communicate their beliefs with expressed confidence proportional to their subjective confidence, determined by the strength of their relevant knowledge or information. Thomas and McFadyen described this as a social norm, but it may be an automatic or even an instinctive pattern of behavior. Whatever its origins or nature, it is essential to the confidence heuristic, because the heuristic works only if most people behave in that way. It facilitates the efficient exchange of information because, in situations of common interests and asymmetric information, people benefit by being persuaded by others with more reliable information than themselves. If people do, in fact, tend to behave in this way, then an interesting psychological question is whether everyone does so to the same degree. For example, the discrepancy between expressed confidence based on strong versus weak information may depend on individual differences such as gender or assertiveness, and the type of decision task may also be a relevant factor.

With regard to the type of decision task, Thomas and McFadyen (1995) suggested that the heuristic applies particularly to tasks in which each person’s information about the available alternatives cannot be communicated directly, so that information strength must be conveyed through the manner in which it is expressed. As Bonaccio and Dalal (2006) pointed out, the choice of a task for this kind of research plays an important role. Different tasks, such as intellective, judgmental, or perceptual, have different properties. Van Swol and Sniezek (2005) identified confidence, out of five factors, as the only predictor of advice utilization on an intellective task. However, in intellective tasks, there are circumstances in which argument quality can overcome expressed confidence: a person who knows the correct answer can sometimes convince others with persuasive arguments even without expressing high confidence (Trouche, Sander, & Mercier, 2014). For intellective tasks, confidence as well as arguments may be relevant, but the confidence heuristic should be most influential in judgmental tasks, where arguments are not easy to formulate. The research reported in this article therefore focuses on judgmental tasks.

No experimental tests of the integrated confidence heuristic have been published to date, and earlier experiments failed to investigate the heuristic as a game-theoretic concept that applies to interactive decision making, treating aspects of it as phenomena of individual decision making. Furthermore, previous research has focused chiefly on the confidence–persuasiveness aspect of the heuristic, omitting the knowledge–confidence aspect. Results have confirmed a general sensitivity of decision makers to communicator confidence (Yates, Price, Lee, & Ramirez, 1996). For example, Tenney, Spellman, and MacCoun (2008) found that people usually give communicators the benefit of the doubt, and presume that confidence reflects accuracy, but revise their judgments if feedback reveals communicators to be poorly calibrated and therefore untrustworthy. Similarly, when groups rather than individuals take decisions, it has been shown that groups are disproportionately influenced by the opinion of the most confident group member (Bloomfield, Libby, & Nelson, 1996), even if this opinion is incorrect. Recent research by Bang et al. (2017) showed further that group members aimed to improve internal communication and decision making by intuitively matching their statements of confidence to a common metric.

Further relevant research has been reported by Bahrami et al. (2010), who studied a low-level perceptual decision-making task to determine whether probability distributions arising from sensory modalities could be communicated. Bahrami et al. investigated communication in dyads to determine a joint answer, and dyads were given accuracy feedback. Their research used individual differences in sensitivity to visual contrast to establish whether communication leads to beneficial joint decisions. They found that communication was necessary to improve collective decision making, but that objective feedback about accuracy was not. They concluded that “human-to-human interpersonal communication is adequately rich to permit sharing of subjective estimates of confidence, and humans are adequately perceptive to make optimal use of this information” (Bahrami et al., 2010, p. 1084). Our research develops this further, seeking to discover how people communicate confidence and what factors influence who is most persuasive. Our research differs from the earlier work insofar as our dyads made individual, not group, decisions and were therefore free to disagree with each other, although there were incentives for agreement.

The confidence–persuasiveness aspect of the heuristic has been confirmed in both intellective and judgmental tasks, in relation to questions with and without objectively correct answers and especially among decision makers with inadequate expertise (Zarnoth & Sniezek, 1997). In simulated job interviews, confidence has been found to be a more persuasive self-presentational strategy than modesty (Tenney & Spellman, 2011), and people making stock market choices have been shown to prefer overconfident financial advisors (Price & Stone, 2004), although feedback demonstrating their overconfidence can reduce their persuasiveness (Sah, Moore, & MacCoun, 2013). However, all of these studies have focused on individual rather than collective decision making.

Research on confidence–persuasiveness effects has also focused on individual judgments or decisions using the Judge-Advisor System (e.g., Price & Stone, 2004). When Price and Stone used the term *confidence heuristic*, they were unaware of Thomas and McFadyen’s (1995) definition, but they tapped into essentially the same idea, namely that more confident advisors are generally assumed to be making more categorically correct judgments and to be more knowledgeable. Research like theirs has typically involved hypothetical decisions in imaginary scenarios without financial incentives, and the results suggested that decision makers’ task-specific confidence could be important, together with demographic and personality differences (notably gender and assertiveness) and the communication channel (Bower & Pulford, 2013; Guadagno & Cialdini, 2002).

Although it may seem intuitively obvious that people will have higher confidence when they have better evidence, there is a substantial literature showing that people are overconfident in some judgments and underconfident in others, and that there are individual differences in confidence. People differ in meta-cognitive introspective ability to estimate the reliability of their own decisions, and this may relate to differing gray matter volume in the anterior prefrontal cortex and reciprocal projections to and from that area (Fleming, Weil, Nagy, Dolan, & Rees, 2010). Recent research indicates that confidence may initially be high after a choice, and some people are initially biased toward rating their confidence high, but then postdecisional processing over time corrects this when confidence judgments and choices are made in different states of mind (Yu, Pleskac, & Zeigenfuse, 2015).

Our experiments included improved study designs characterized by real interactive decisions with financial incentives, enabling both the knowledge–confidence and the confidence–persuasiveness aspects of the confidence heuristic to be investigated in situations of common interests. More specifically, we report the first comprehensive test of the integrated confidence heuristic across three experiments using different judgmental tasks in common-interest games with asymmetric information. We tested the heuristic with two different stimulus classes, namely faces (Experiment 1) and geometric shapes (Experiments 2 and 3); in all three cases, we predicted that pairs of decision makers with asymmetric information would succeed in coordinating on the target response. Further, in Experiment 3 we examined whether the effectiveness of the confidence heuristic depends on the medium or channel of communication. Specifically, because so much interpersonal communication now takes place through electronic devices, we compared face-to-face (FtF) communication with computer-mediated (CmC) communication (instant messaging). We predicted that dyad members would make better decisions when communicating face-to-face than through instant messaging, because only FtF communication includes a full range of nonverbal information (facial expression, gestures, tone of voice, and so on) that may be important in signaling confidence and thereby enabling the confidence heuristic to operate.

## Experiment 1

Our first experiment set out to investigate the confidence heuristic in interactive decisions through the use of a facial recognition task. In line with previous research on eyewitness identification (e.g., Tenney, MacCoun, Spellman, & Hastie, 2007, 2008), we designed a task requiring the identification of a suspect from a set of photos on the basis of evidence from an electronic facial identification technique (E-FIT) of a target person. We used faces as stimuli because it is difficult to explain purely verbally why a facial resemblance seems strong or weak. The confidence heuristic applies to situations (common in everyday life) in which there exists a target choice that would be optimal for both individuals involved, but they are not certain which it is, although one individual has stronger evidence pointing to it than does the other. Hence, weak-evidence pair members who choose faces that most closely resemble their weak-evidence E-FITs are not choosing the option that would be best for themselves and their partners, even if these faces appear best from their own weak-evidence perspectives.

Pairs of participants were asked to make their decisions while one was provided with strong evidence (a good E-FIT likeness of the target) and the other weak evidence (an E-FIT lacking strong resemblance to any of the faces). The pair members were permitted to communicate verbally while both looked at the same set of suspect photos on each trial, without seeing each other’s E-FIT evidence. We tested the hypotheses that evidence strength would affect task-specific confidence and that higher confidence would in turn result in increased persuasiveness, leading to better decisions by both pair members. To monitor the possibility of other factors overriding or enhancing the confidence heuristic, we examined factors that have been shown to influence persuasiveness in communication. We measured assertiveness in case a lack of assertiveness causes some participants to give in to the other pair member even if the other has weak evidence. We measured need for closure and need for cognition to determine whether people who just want to close the decision and reduce their uncertainty, or those who do not want to think deeply about the evidence provided to them, are less influenced by the confidence heuristic and give in to the pair member with weaker evidence. Finally, we investigated effects of age and gender to determine whether older participants are better at using the confidence heuristic because of experience and whether women are better than men at detecting nonverbal cues of confidence.

### Method

#### Participants

The participants were 56 undergraduate students (28 women and 28 men) with a mean age of 22.09 years (*SD* = 4.83), who were recruited through posters displayed around the university campus. Payments depended on their own and their partner’s decisions in the task. After they completed the experiment, payoffs across all 23 trials were summed for each individual, and the totals were paid out (*M* = £7.44, approximately US$9.54). We aimed for 50 participants to detect a small to medium effect size (0.3) with 80% power. We terminated data collection after reaching our target sample size, without having conducted any preliminary analyses before stopping. We have reported all measures and conditions, and no data were excluded from the experiments reported in this article. Ethical approval for all three studies was granted by the University of Leicester Psychology Research Ethics Committee.

#### Design

Using a repeated-measures design, we manipulated the evidence strength (strong or weak), aiming to induce either high or low confidence in each pair member on each trial. The first main dependent variable was self-reported confidence (on a scale ranging from 0 to 100) following each trial, with separate mean confidence scores calculated for trials with strong and weak evidence.

A second dependent variable was persuasiveness—the percentage of trials on which pair members successfully persuaded their partners. We had estimates from a pilot study confirming that, in the absence of consultation, strong-evidence pair members would normally choose the target faces that their E-FITs were designed to resemble, and we interpreted the percentage of agreements by weak-evidence pair members with these target choices as a rough-and-ready index of persuasion by the strong-evidence partner, although there remains a 1 in 9 probability that the weak-evidence partner chose it by chance, and not as a result of persuasion, because there were nine faces from which to choose. The degree of agreement on the target faces was an indication of how good the decisions were and was thus our measure of performance. We scored agreement by both pair members on any of the other faces as persuasion by the weak-evidence pair member, not only because the pilot data suggested that, without consultation with a partner, the strong-evidence pair member would usually have chosen the target face, but also because the probability of the strong-evidence pair member choosing the same face that the weak-evidence partner chose, purely by chance, is only one in nine.

Additionally, we recorded age, gender, gender composition of the participant pairs, closeness of relationship within the participant pairs, assertiveness, need for closure, and need for cognition. Finally, we taped and transcribed the verbal exchanges between pair members in each trial.

#### Materials

**Figure 1 fig1:**
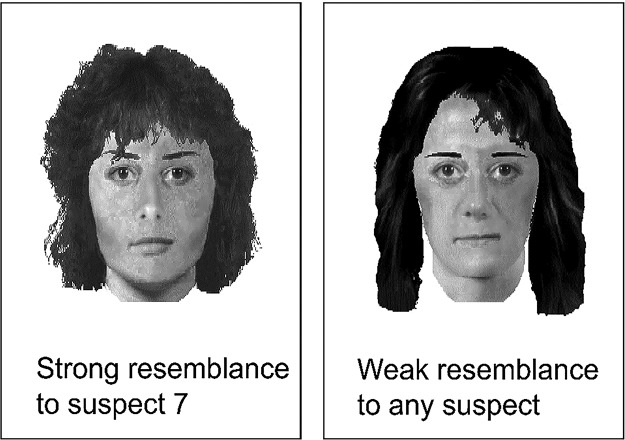
Example E-FITs used in Experiment 1.

All participants received a questionnaire booklet with one practice trial and 23 experimental trials that required them to match an E-FIT with one of nine possible suspect photos.^1^ Example stimuli are displayed in Figure 1. Participants were asked to imagine that two witnesses to a crime had each created an E-FIT of the perpetrator and that each pair member was looking at one of the E-FITs and at the same set of suspect photos to decide which was the target suspect. They were allowed to exchange verbal information but not to see each other’s E-FITs. They were told the following: “Eyewitnesses can often differ from each other because they have different views of the event and the criminal, so the E-FITS created may be good or not-so-good likenesses of the suspect.”[Fig-anchor fig1]

The two booklets for each pair were matched so that whenever one pair member viewed a strong E-FIT of the target, the other viewed a weak E-FIT bearing no strong resemblance to any of the faces. The same E-FITs were used twice, shown once to the first pair member and at a different point in the session to the partner. Because each pair member viewed exactly the same stimuli as the other, but at different times, the data enabled us to determine the number of trials on which the opinion of the pair member with strong evidence was accepted by the partner, controlling for individual differences in facial recognition ability and idiosyncratic differences between photos. Each participant was given an example set of nine suspect photos plus an example E-FIT, followed by eight strong-evidence sets (while the partner had weak evidence), eight weak-evidence sets (while the partner had strong evidence), four fillers in which both pair members had strong evidence, and three fillers in which both had weak evidence, in a randomized order. The fillers were included to conceal the evidence strength manipulation.

All suspect photos were color images of young Caucasian men and women selected from the Psychological Image Collection at Stirling (see http://pics.psych.stir.ac.uk/). The E-FITs were constructed using custom software used by U.K. police forces. Half of the E-FITs were designed to resemble one of the faces strongly and half to lack strong resemblance to any of the photos, although they could be viewed as weak evidence for one or more of the nontarget faces. The degrees of likeness of the E-FITs to the suspect photos and the degrees of confidence that the participants felt in their identifications were evaluated in a pilot study in which 32 participants rated the resemblance of E-FITs to target faces across 73 trials. The materials were calibrated so that pair members with weak evidence would have an accuracy rate near 11% (pure guessing) and low confidence, whereas pair members with strong evidence would reliably select the target alternative with high confidence. The data from the pilot study showed that, when the evidence was weak, participants chose the target faces on 7.04% of trials and with 29.37% confidence; and when the evidence was strong, they selected the target faces on 73.45% of trials with 70.59% confidence (see Table 1). With weak evidence, they chose nontarget alternatives on 92.96% of trials with 37.67% confidence; with strong evidence, they chose nontarget alternatives on 26.55% of trials with 43.78% confidence.[Table-anchor tbl1]

Thus, by designing the pilot study materials carefully, we were able to assemble pairs comprising a weak-evidence E-FIT leading to the target faces being chosen with very low frequency and low confidence and a strong-evidence E-FIT leading to the target faces being chosen with high frequency coupled with much higher confidence. The strong-evidence E-FITs induced mean confidence of 70.59% when target faces were chosen (range = 59%–82%), whereas the weak-evidence E-FITs induced mean confidence of 37.67% when nontarget faces were chosen (range = 31%–47%). The pilot study thus provides a manipulation check confirming not only that we successfully manipulated strong versus weak evidence but also that the strong-evidence E-FITs, when chosen, induced much higher confidence than the weak-evidence E-FITs, without leaving either pair member in complete certainty or complete doubt.

Because assertiveness could affect persuasiveness and decisiveness, we administered the Rathus Assertiveness Schedule (Rathus, 1973), a 30-item Likert-type scale measuring assertive behavior across a variety of contexts, with higher scores indicating greater assertiveness. For analogous reasons, we also administered the Need for Cognition Scale (Cacioppo, Petty, Feinstein, & Jarvis, 1996), designed to measure how much people engage in and enjoy thinking, and the Need for Closure Scale (Kruglanski, Webster, & Klem, 1993), designed to measure their general tendency to prefer certain to uncertain knowledge. Participants also self-rated the intimacy of their relationship with their experimental partners on a six-point scale, comprising 1 (*complete strangers*), 2 (*weak acquaintance*), 3 (*regular acquaintance*), 4 (*friend*), 5 (*good friend*), and 6 (*intimate partner*).

#### Procedure

After giving informed consent, participants received booklets containing experimental materials. In their own time, they read the task instructions, and the experimenter answered any questions. Participants were fully informed about their options and the financial incentives in the task, and they were aware that the E-FIT evidence provided for solving the task would differ between pair members. For each of 23 decisions, participants were told the following: “If you both choose the same face of the person who did commit the crime, you will get 40p (approximately US$0.50) each”; “If you both choose the same but innocent person, then you get 20p (approximately US$0.25) each”; and “If you choose two different people, then you each get nothing, even if one of you chooses the person who is guilty.”

Pair members sat on opposite sides of a table, divided by a low partition to hide their test materials from each other but to allow face-to-face communication, and a voice recorder was switched on. For each of the 23 experimental trials (including fillers to conceal the evidence strength manipulation), pairs were allowed up to 2 min to discuss which face, from an identical array of nine suspect photos given to both, looked most like the face portrayed in the E-FIT and to record their individual decisions. After circling their decisions on their answer sheets, participants rated how confident they felt that they had chosen the correct answer on a scale, ranging from 0 (*not at all sure that I chose the right person*) to 100 (*completely sure that I chose the right person*). After completing all 23 trials, the payoffs earned in each trial were calculated and paid.

### Results

#### Quantitative results

A manipulation check confirmed that evidence strength had a significant effect on confidence: participants reported higher confidence in their decisions when they received strong evidence (*M* = 69.69, *SD* = 11.04) than when they received weak evidence (*M* = 57.21, *SD* = 12.65), *F*(1, 54) = 51.366, *p* < .001, (partial η^2^ = .49, BF_10_ > 100), and there was no significant effect of gender (BF_10_ = 0.24; see Footnote 1). Here and elsewhere, BF_10_ refers to the Bayes factor likelihood of the data under the experimental hypothesis relative to the null hypothesis, calculated using the software package JASP Team (2016). Although pair members who received weak evidence reported significantly lower confidence, their confidence was not very low, because by the time they made their decisions, they had discussed the alternatives with their experimental partners who had strong evidence, and this presumably boosted their own confidence, because they were usually persuaded by their partners.

On most trials (60.94%), the pair members with strong evidence persuaded their partners with weak evidence to agree with their decisions. The pairs disagreed with each other and chose different alternatives infrequently (*M* = 7.14% of trials, *SD* = 12.58). The persuasions were significantly more frequent under the effect of strong evidence (*M* = 60.94%, *SD* = 22.24) than under weak evidence (*M* = 31.92%, *SD* = 17.18), *F*(1, 54) = 56.112, *p* < .001, (partial η^2^ = .51, BF_10_ > 100), and once again there was no significant effect of gender (BF_10_ = 0.27). No significant correlations were found between persuasiveness, confidence, and any of the individual difference variables (age, gender, relationship with experimental partner, assertiveness, difference between assertiveness levels in the pair, need for cognition, need for closure; all *p*s > .05). A Bayesian repeated-measures analysis of variance (ANOVA) showed that none of the individual differences influenced persuasion significantly (all BF_10_ < 1.0).

Table 1 allows for a comparison of choices in Experiment 1 and in the pilot study (without consultation between pair members). The top panel of Table 1 shows data for eight weak-evidence E-FITs and in the bottom panel data for eight E-FITS providing strong evidence for one of the faces in the line-up. In Experiment 1, we can see that most pair members with strong evidence chose the target face (66.07%), and slightly fewer of those with weak evidence were persuaded by their strong-evidence partners to choose the target face (60.49%). The discrepancy between the two means is due to the fact that pair members were allowed to disagree with each other and choose different answers, and they did so on a small number of occasions.

Table 1 also shows that whereas pair members with weak-evidence E-FITs chose the target faces on 60.49% of Experiment 1 trials, after they had discussions with their strong-evidence partners, participants in the pilot study chose those faces on only 7.04% of occasions. This is clear evidence for the persuasive power of the strong-evidence, high-confidence pair members in Experiment 1. In the pilot study, participants with strong evidence were able to identify the target face correctly on 73.45% of trials, but this figure dropped slightly to 66.07% after they had discussions with weak-evidence partners in Experiment 1, suggesting that they were sometimes (on about 7% of trials) swayed away from the target choice. The purpose of this analysis is to provide an estimate of what participants would have chosen prediscussion, without having to ask them explicitly. We wished to avoid asking pair members in this experiment to commit to initial decisions before conferring with their partners, because eliciting initial judgments could cause a confound by introducing anchoring effects (Olson & Stone, 2005; see also Gawronski, Bodenhausen, & Becker, 2007), and elicitation of prediscussion choices would thus have tended to reduce any persuasion effect in the experiment.

Finally, we checked if there were any changes in the dependent variables over time, to discover whether task experience altered behavior. Across four trial blocks of four faces, in which pair members invariably had different evidence strengths, the number of agreements with the pair member with strong evidence remained practically stable, but we found differences in confidence. Because the same task materials (faces) were used twice, once in the first two trial blocks and once in the second two, observed changes in confidence cannot be explained by idiosyncratic differences between task materials in different trial blocks. The data showed that after initial stability in the first three trial blocks, there was a small drop in confidence in the last trial block for pair members with both strong evidence (6% lower) and weak evidence (8% lower). From this we conclude that while the efficacy of the confidence heuristic did not change over time, the difficulty of the task began to take its toll on confidence toward the end.

Taken together, these results indicate that the pair members with stronger evidence were significantly more confident and more often persuasive than those with weak evidence. This provides support for both aspects of the confidence heuristic. Not only was the confidence heuristic effective, but it appears to have had far more influence on decision making than any individual difference variable that we investigated.

#### Qualitative results

We examined the verbal transcripts for task comprehension, and we classified typical contents in the communications, using a taxonomy of confidence cues provided by Wesson and Pulford (2009), according to the way in which participants voiced their confidence. We observed that participants gave explicit confidence judgments to each other and asked for clarification on how confident/sure/certain the other pair member was. To indicate their degrees of confidence, they said things such as, “I’m not sure,” “I’m mostly sure,” “I’m pretty sure,” “I’m as sure as I can be,” and “I’m quite sure.” An example of a highly confident statement is the following:

I’m pretty sure mine’s Number 3. It’s quite identical; the ears are quite long on my picture, the hair is practically identical to Number 3’s, er . . . I have the same eyebrows and the same mouth and the same expression. Mine’s practically Number 3. . . . I’m quite happy to put down. . . 100% confident, it’s practically, yeah, I’m quite certain that its Number 3.

In contrast, the following is an example statement indicating low confidence: “I find this very difficult. I have no idea, to be very honest.” Several statements provided qualitative support for the persuasiveness of high confidence shown in the quantitative data. For example: “I’m very dubious about this, but I don’t think he looks like anybody to be very honest, so I would tend to go with what you said, so Number 7.” In some conversations it was clear that pair members could not decide on an answer and were thus happy to go along with their more confident partners. In other situations, weak-evidence pair members with some degree of confidence were persuasive and their strong-evidence partners backed down, perhaps for the sake of group harmony. Pair members sometimes checked how confident their partners felt with questions such as, “Are you sure?” “If you’re quite sure,” “Really?” For example, the conversation between Participant 29 and Participant 30 included the following exchange: “Errm, I mean, you wanna give a percentage about how sure you are about 7 because I would say. . . .” “I’d go for near 75 to 80.” “That’s a lot higher than me, so let’s go 7.”

In most cases, participants chose the first answer suggested, these cases outnumbering by around 2.5:1 cases where the first answer was not chosen in the end. It appeared that pair members with strong evidence would often identify the matching face in the array but on some occasions be swayed from their choice by their partners, as their confidence declined over the conversation. Getting in with the first suggested answer seemed to have an important psychological effect; for example, one pair member with weak evidence began the conversation with the following: “Straight away I’d say . . .”; but this participant was interrupted at that point by the partner with strong evidence jumping in and saying “3” before the first pair member’s choice could be voiced. A large proportion of the verbal exchange involved pair members describing their own E-FITs and comparing particular features with their partners’ E-FIT descriptions. Some participants attended to minute details: “Er . . . well if you look at Number 4, you can just make out in the corner of the E-FIT a little stud which could be an earring, ‘cos Number 4’s wearing sort of square earrings.” Consequently, many pairs were cut off prematurely by the 2-min time limit on verbal discussion.

Overall, the verbal protocols confirm that the facial identification task provided a useful decision context for testing the confidence heuristic. But the facial stimuli also elicited excessively detailed discussions, including minute descriptions and comparisons of individual facial features, and this sometimes crowded out and overshadowed the type of comments that are the focus of our investigation, namely those primarily reflecting or expressing degrees of confidence. A related problem was that the facial identification task proved very difficult. Many participants struggled with it, and even pair members with strong evidence occasionally failed to recognize the target face from the E-FIT and thus argued for nontarget alternatives. As a consequence, even when they succeeded in persuading their partners, they may have persuaded them to choose nontarget alternatives, thus earning lower persuasiveness scores and incentive payments than they could have achieved. Furthermore, when strong evidence was not strong enough to induce high confidence, they may have failed to argue for the target face altogether. Overall, the difficulty of the facial identification task may thus have masked the potential strength of the confidence heuristic. To address this problem, we designed a second experiment using simpler stimuli (geometric shapes) and an easier decision task with two rather than nine response alternatives.

## Experiment 2

Experiment 2 was designed to replicate Experiment 1 using a simpler class of stimuli and an easier decision task. We chose elementary geometric shapes that pair members tried to match for size because we wanted to avoid the detailed discussions about individual stimulus features that had occurred in Experiment 1 and, thus, to allow more time for confidence-signaling communication. In addition to the three personality measures used in Experiment 1, we added a measure of dogmatism because Davies (1998) reported it to be related to higher confidence in judgments, with dogmatic/authoritarian individuals showing more information-processing bias toward supporting evidence and against contradictory evidence.

### Method

#### Participants

We tested 82 participants (35 men and 47 women) with a mean age of 19.83 years (*SD* = 2.90), recruited as in Experiment 1. Out of 41 participant pairs, 21 were mixed gender, 13 were all female, and seven were all male. All participants provided informed consent. Once again, participants received payments depending on their own and their experimental partners’ choices. On each trial, if both pair members coordinated by choosing the target shape to which the strong evidence pointed, then each received 40p (US$0.50); if both chose the same but nontarget shape, then each received 20p (US$0.25); if they chose different shapes, then each received nothing. The payoffs from all trials were added together for the final payments (*M* = £5.64; approximately US$f7.23). Considering the large effect size in Experiment 1, and the number of predictors in the regression, we aimed for a minimum of 70 participants to detect a large effect size (0.4) with 95% power. We terminated data collection before performing any preliminary analyses.

#### Design

The design was identical to Experiment 1. Only the stimuli and materials differed, as described below.

#### Materials

The test materials were selected from 30 shapes in a pilot study, in which 26 participants attempted to choose which of two shapes was closest in size (undefined) to a target shape and rated their confidence in their answers. The materials were calibrated so that pair members with weak evidence would have an accuracy rate near 50% (pure guessing) and low confidence, whereas pair members with strong evidence would reliably select the target answer and have high confidence. The data from the pilot study showed that, when the evidence was weak, participants chose the target shape on 53.00% of trials (essentially guessing) with 71.67% confidence. When the evidence was strong, they chose the target shape on 90.50% of trials with 87.83% confidence. With weak evidence, they chose the nontarget alternative on 47.00% of trials with 75.83% confidence; and with strong evidence, they chose the nontarget alternative on 9.50% of trials with 67.00% confidence. The latter result shows that although the vast majority found the task easy when given strong evidence, a few strong-evidence pair members found it hard to select the closest-size shape.

**Figure 2 fig2:**
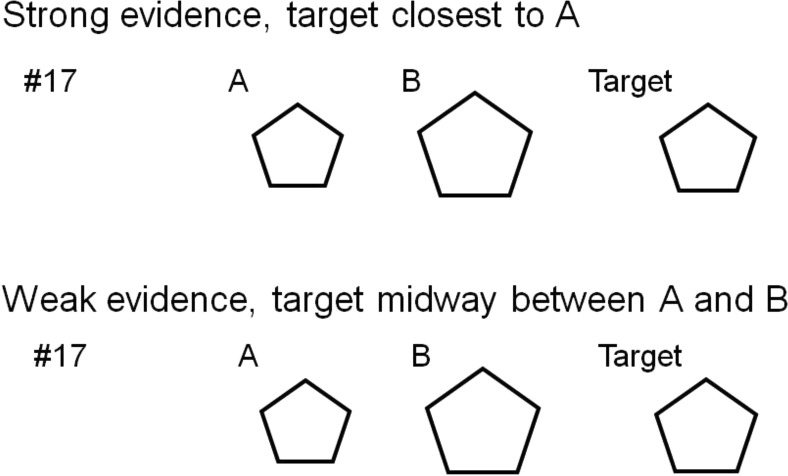
Example stimuli used in Experiments 2 and 3.

Both pair members received paper folders containing two pages of instructions followed by a separate page for each decision trial (12 test trials and 4 filler trials). Additionally, participants were given a separate answer sheet to record their decisions. On each trial, they were presented with one target shape and two test shapes (see Figure 2). Both pair members were shown the same two test shapes on each trial but were given different target shapes. The pair members did not know that they had slightly different target shapes. The target shapes were either close in size to one of the two test shapes (strong evidence) or midway between the sizes of the two test shapes (weak evidence).[Fig-anchor fig2]

The task involved identifying the test shape closest in size to the target shape and, if possible, agreeing on the same shape with the other pair member. Participants were incentivized with financial rewards depending on their own and their partners’ choices in the experiment, essentially as in Experiment 1.

We used eight different geometric shapes, each presented to every participant pair twice. The order of trials and the order in which participants were presented with either strong or weak evidence were randomized, but each participant completed the same numbers of trials with strong and weak evidence. The 20-item short-form Rokeach Dogmatism Scale (Troldahl & Powell, 1965) was given to participants along with the three scales mentioned in Experiment 1.

#### Procedure

The procedure used in Experiment 2 was almost identical to Experiment 1, the main difference being the reduced number of 16 experimental trials to lessen boredom.

### Results

The manipulation of evidence strength once again had a significant effect on confidence. When pair members received strong evidence, they reported significantly higher confidence levels (*M* = 83.89, *SD* = 10.22) than when they received weak evidence (*M* = 67.66, *SD* = 16.44); *F*(1, 80) = 101.428, *p* < .001, (partial η^2^ = .56, BF_10_ > 100), and there was no significant effect of gender on confidence (BF_10_ = 0.32).

On the vast majority of trials (85.77%), the pair members with strong evidence persuaded those with weak evidence to agree with their decisions. The pairs failed to reach agreement infrequently (*M* = 6.30% of trials, *SD* = 9.76). The number of persuasions under the effect of strong evidence (*M* = 85.77%, *SD* = 14.61) was significantly higher than under weak evidence (*M* = 7.93%, *SD* = 12.35); *F*(1, 80) = 1226.505, *p* < .001 (partial η^2^ = .94, *d* = 3.948, BF_10_ > 100), and once again there was no significant effect of gender (BF_10_ = 0.18). Furthermore, persuasiveness and confidence were not significantly affected by any of the individual difference variables (age, gender, relationship with experimental partner, assertiveness, need for cognition, need for closure, dogmatism, difference between assertiveness or dogmatism levels in the pair (all *p*s > .05; all BF_10_ < 1.0).

Experiment 2 corroborates the findings of Experiment 1, providing clear evidence for both aspects of the confidence heuristic in an entirely new domain of judgment and suggesting that evidence strength and resulting confidence have more influence on joint decisions than any of the individual differences that we investigated. Compared with a facial identification task used in Experiment 1, the shape task of Experiment 2 improved overall task performance, with much higher agreement on the target answer, and increased the size of the confidence heuristic effect.

## Experiment 3

Experiment 3 used the same shape stimuli as Experiment 2 but extended the previous investigation by comparing the effect through two different communication channels: face-to-face (FtF) and computer-mediated communication (CmC), because this is a factor that has been found to influence persuasiveness in individual decisions (Guadagno & Cialdini, 2002). The effect of communication channel on the confidence heuristic has important implications for the persuasiveness of e-mail, Facebook, Twitter, and text message communications, and whether the heuristic can moderate the diffusion of ill-informed and false information through such forms of communication. There are grounds for expecting the confidence heuristic to be attenuated or eliminated in CmC, because nonverbal cues, including movement (kinesics), facial expressions, gestures, and tone of voice, are all filtered out, and these may be crucial for communicating confidence.

We examined gender differences once again, because the judgment task in this experiment is more quantitative than the task in Experiment 1, and Lee (2005) showed that men are more likely to be persuaded by a communicator’s high confidence when it is expressed numerically, whereas women are more likely to be persuaded when it is expressed verbally. We included a measure of trait dominance, because Anderson and Kilduff (2009) showed that dominant people have higher influence in groups and also are rated as more competent, hence dominance may be more important for persuasiveness than confidence. Additionally, we assessed a new range of individual difference variables, namely the Big Five (Gosling, Rentfrow, & Swann, 2003) and judgmental self-doubt (Mirels, Greblo, & Dean, 2002). We included judgmental self-doubt (JSD), because people’s lack of trust in the accuracy of their own judgments may make them less persuasive and hence more susceptible to persuasion. This suggests that we might expect to observe low persuasiveness in participants with high JSD scores in the strong-evidence condition.

Finally, we examined verbal timing effects. Gabbert, Memon, and Wright (2006) reported that, in pairs of witnesses remembering an event, the pair member who spoke first was usually more persuasive. Bang et al. (2014) suggested, based on the finding that confidence estimates are negatively correlated with their reaction times (Patel, Fleming, & Kilner, 2012; Pleskac & Busemeyer, 2010), that faster judgments may be interpreted as indicating more confident and thus more accurate information. Weak evidence may make people hesitant to suggest an answer, so that the more confident pair members with strong evidence may tend to suggest an answer first. In this experiment, we therefore recorded both who spoke first and who suggested an answer first. By comparing verbal timing effects in FtF and CmC conditions, we can determine if they need to be supplemented by other cues, available in FtF but not in CmC, to be effective for persuasion and coordination.

### Method

#### Participants

The sample consisted of 78 undergraduate psychology students (54 females and 24 males) with a mean age of 19.39 years (*SD* = 2.20), recruited as in Experiments 1 and 2 and randomly assigned to 39 participant pairs for testing. Of these, 26 pairs contained two female participants, 11 two male participants, and two mixed genders. We aimed for the pairs to be same gender in case talking with someone of the opposite sex inhibited conversation. All participants provided informed consent. All participants received course credits for taking part; additionally, every participant was paid according to incentive earnings from the decision task (*M* = £3.54; approximately US$4.54). Considering the large effect size in Experiments 1 and 2, two conditions, covariates, and financial cost, we aimed for 75 participants to detect a large effect size (0.5) with 80% power. We terminated data collection before performing any preliminary analyses.

#### Design

The experiment used a 2 (Channel) × 2 (Evidence Strength) mixed model design. Participant pairs were randomly assigned to two different communication channel treatment conditions (between-subjects), either FtF or CmC. Additionally, evidence strength (within-subjects) was manipulated as in Experiment 2. The experiment included six trials with strong evidence and six with weak evidence. The main dependent variables were again persuasiveness and self-reported confidence. To determine whether confidence is communicated by timing of verbal comments, we calculated the percentage of trials on which each pair member spoke first and the percentage of trials on which each pair member suggested an answer first. Finally, we measured several demographic and individual difference variables, namely gender, gender composition of the participant pair, judgmental self-doubt, trait dominance, and the Big Five personality traits.

#### Materials

Participants were given paper folders with instructions and decision trials, similar to those used in Experiment 2. Additionally, several paper-based questionnaires were distributed, and participants completed these in random order after finishing the interactive decision task. The questionnaires included a Judgmental Self-Doubt scale (Mirels et al., 2002), three items measuring trait dominance (dominance, assertiveness, and forcefulness) from the Revised Interpersonal Adjective Scales (IAS-R; Wiggins, Trapnell, & Phillips, 1988), and the Ten Item Personality Inventory (Gosling et al., 2003).

#### Procedure

In the FtF condition, the procedure was identical to Experiments 1 and 2. Pair members sat opposite each other with a low partition that enabled face-to-face, verbal conversation but shielded their test materials from each other’s view. In the CmC condition, pairs met briefly and were immediately led into separate rooms; they could not see or hear each other, but written communication was enabled via Slack, an online instant-messaging program. The experimenter gave a signal whenever pair members were required to turn over the page and begin a new decision trial. In this experiment, pairs had up to three minutes to discuss each problem and record their decisions on their individual answer sheets.

After completing the required decisions, one decision trial was drawn at random by the experimenter to determine incentive payments. Both pair members received the financial rewards earned on the selected trial. This incentive system is designed to avoid the use of averaging strategies across experimental trials and to ensure that participants treat every trial as a separate decision task that might determine their final payoffs; there is experimental evidence that it achieves this (for discussions of this incentive system, see Bolle, 1990; Cubitt, Starmer, & Sugden, 1998).

### Results

**Figure 3 fig3:**
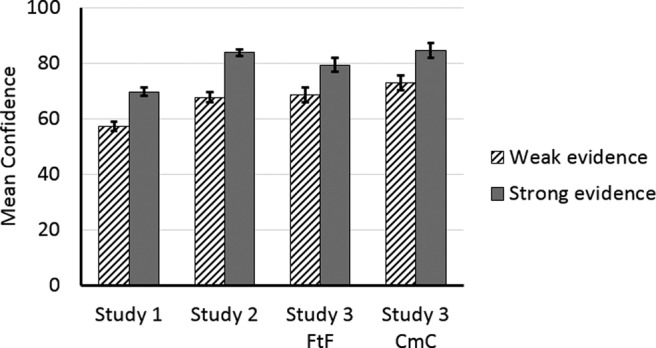
The influence of weak and strong evidence on mean confidence in all three experiments, with standard error bars.

The manipulation of evidence strength had a significant effect on individuals’ confidence: a mixed ANOVA revealed that when pair members received strong evidence, they reported significantly higher confidence levels (*M* = 81.91, *SD* = 16.13) than when they received weak evidence (*M* = 70.67, *SD* = 15.88), *F*(1, 72) = 34.425, *p* < .001, (partial η^2^ = .323, BF_10_ > 100). As shown in Figure 3, strong evidence promoted confidence in all three of our experiments. Communication channel (FtF vs. CmC) did not significantly affect confidence, *F*(1, 72) = 2.177, *p* = .144, (BF_10_ < 1.0), nor did the interaction of evidence strength and communication channel, *F*(1, 72) = 0.055, *p* = .816.[Fig-anchor fig3]

**Figure 4 fig4:**
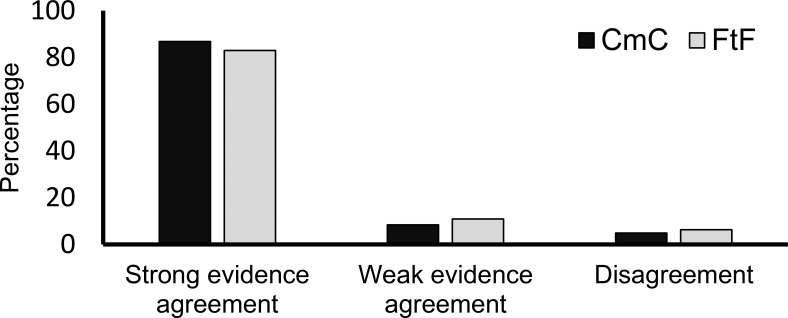
Percentages of trials in Experiment 3 in which pair members agreed on the alternative associated with strong evidence, agreed on an alternative with weak evidence, or disagreed.

As is clear in Figure 4, on the vast majority of trials (84.83%), pair members with strong evidence persuaded those with weak evidence to agree with their choices. A two-way mixed-design ANOVA was performed to check whether persuasiveness (as measured by the number of trials on which a pair member persuaded the partner) differed depending on evidence strength (strong or weak) or on communication channel (FtF or CmC). Evidence strength was found to have a significant main effect: the percentage of persuasions was significantly higher under the effect of strong evidence (*M* = 84.83%, *SD* = 19.77) than under weak evidence (*M* = 9.62%, *SD* = 15.55); *F*(1, 76) = 562.592, *p* < .001, (partial η^2^ = .881, BF_10_ > 100). Surprisingly, communication channel had no significant effect, *F*(1, 76) = 0.081, *p* = .776, (BF_10_ < 1.0), nor did the interaction of evidence strength and communication channel, *F*(1, 76) = 1.026, *p* = .314.[Fig-anchor fig4]

Taken together, these results provide further evidence for the confidence heuristic and extend it across FtF and CmC channels. Contrary to our predictions, we did not find any significant effects of communication channel, with FtF and CmC yielding remarkably similar effects of knowledge on confidence and confidence on persuasiveness. This suggests that it is verbal rather than nonverbal communication of confidence that drives the confidence heuristic, although timing of verbal communication was obviously a significant signal of confidence, as explained in the following text.

We carried out two ANOVAs, with communication channel (FtF vs. CmC) and gender as between-subjects factors and evidence strength (strong vs. weak) as a within-subjects factor, on both the percentage of trials on which pair members suggested an answer first and the percentage of trials on which they spoke first. Evidence strength was the only significant factor: when pair members had strong evidence, they suggested the answer first on 73.03% (*SD* = 23.29) of trials, significantly more frequently than the 26.97% (*SD* = 23.25) when they had weak evidence, *F*(1, 72) = 114.605, *p* < .001, (partial η^2^ = .614). Communication channel and participants’ gender did not yield significant effects. The second ANOVA showed that pair members spoke first more frequently in the strong-evidence than the weak-evidence condition (*M* = 63.82%, *SD* = 26.58 and *M* = 36.18%, *SD* = 26.58, respectively), *F*(1, 72) = 42.909, *p* < .001, (partial η^2^ = .373), and there was no evidence of any gender or communication channel effects.

The verbal protocols revealed that speaking first but not stating an answer often signaled low confidence and typically took the form of a question or prod, encouraging the partner to speak (e.g., “So which one do you think?”) or just stating something unrelated to the decision (e.g., “So, Task 5”). The verbal protocols also revealed that the conversations were very short in this experiment and lacked details or much content. For example, dyad 38’s conversation on shape 6 was typically minimal, starting with Player A saying “A?” followed by a reply from Player B of “A,” and Player A concluding, “Yeah.” Comments that were a bit longer included remarks such as: “It’s just a little smaller,” “B looks slightly bigger,” “They’re both really similar,” “I reckon it’s A too. How confident are you?” Common phrases were “I think it’s A [or B],” “I reckon it’s A [or B],” or just a suggested answer with or without a questioning tone.

We performed a multiple linear regression analysis, ignoring communication channel (because of a small *n*), to see which if any individual-difference variables predicted persuasiveness. In the strong-evidence condition, no significant predictors were found. In the weak-evidence condition, openness (β = −4.562, *p* = .002, importance = 0.563) and trait dominance (β = 3.582, *p* = .033, importance = 0.267) predicted the percentage of persuasions. Thus, participants who were more dominant and less open were more persuasive when they had weak evidence. We performed a similar analysis to assess the predictors of participants’ confidence in their decisions. When evidence was strong, mean confidence was predicted only by the percentage of persuasions (β = 0.271, *p* = .005, importance = 1.000), and when evidence was weak, there were no significant predictors.

Correlation analysis showed that, when there was strong evidence, the frequency of persuasions correlated significantly with how frequently the participant suggested an answer first (*r* = .404, *N* = 76, *p* < .001), and a similar correlation was found in the weak-evidence condition (*r* = .434, *N* = 76, *p* < .001). In both conditions, persuasions were nonsignificantly related to how often a pair member spoke first. Thus, it appears that giving an answer first is a crucial factor increasing persuasiveness, both when the evidence is strong and when it is weak. It is striking that none of the personality measures that we investigated correlated with how frequently a pair member suggested an answer first. However, in the strong-evidence condition only, participants who had higher dominance scores spoke first more frequently (generally not suggesting an answer; *r* = .310, *N* = 76, *p* = .006).

## General Discussion

This article reports the first empirical evidence for the integrated confidence heuristic, replicated across three experiments, operating through face-to-face and computer-mediated communication. In our experiments, pair members with strong evidence tended to be more confident and more persuasive than their partners with weak evidence, confirming the complete knowledge–confidence–persuasiveness causal path implied by the heuristic in common-interest games with asymmetric information. These findings may seem somewhat surprising in the light of robust evidence that people tend to weight their own judgments more highly than those of others (Yaniv, 2004). In the light of that evidence, it is far from obvious that people will go along with the opinions of their more confident partners. However, subsequent research (Rader, Soil, & Larrick, 2015) has shown that people who receive advice from others before forming their own judgments tend to push away from the advice, whereas those who form independent judgments before receiving advice are more likely to adjust in the direction of the advice. In our experiments, and more generally in circumstances in which the confidence heuristic is hypothesized to occur, individuals form independent judgments before receiving advice, and that may explain why they do not appear to ignore the opinions of their sometimes better informed and more confident partners.

Compared with previous research on the confidence–persuasiveness path, our experiments included much improved materials pretested in pilot studies. They also involved participants making interactive decisions with real outcomes and financial incentives, not merely individual decision making in hypothetical scenarios. The repeated-measures design controlled for individual differences influencing the results. Our findings accord with those of previous studies on the confidence–persuasiveness effect in individual decisions (e.g., Price & Stone, 2004; Sah et al., 2013; Tenney et al., 2007, 2008), but we also show that strong evidence increases confidence before confidence increases persuasiveness, and that in decision-making pairs with shared goals and asymmetric information, the heuristic helps pair members to coordinate on optimal joint decisions.

Experiment 1, in which we used a facial identification task with nine response alternatives, yielded the lowest percentage (61%) of optimal joint decisions. In Experiment 2, we therefore used an easier decision task involving size judgments of simple geometric shapes with just two response alternatives, and this yielded 85.77% optimal joint decisions, replicating the main findings of Experiment 1 with a very large confidence–persuasiveness effect size (Cohen’s *d* = 3.94). In Experiment 3, using the same decision task as Experiment 2, the confidence heuristic generated 84.83% of optimal joint decisions. The confidence heuristic effect is clearly powerful and robust when people are motivated by common interests. There were clearly some instances where people failed to communicate their confidence persuasively enough, resulting in suboptimal agreement or disagreement, and these instances deserve further investigation by future researchers.

Using two different tasks was beneficial, because it has allowed us to show that when people cannot easily articulate reasons for their choices, for example when judging the size of something, they are more reliant on the confidence heuristic to decide who is most likely to be making the best judgment. In contrast, when there is more to discuss and reasons can be articulated more easily, for example about mouth shape or hairstyle in our facial judgment task, the confidence heuristic functions less efficiently. The change in stimuli from faces to shapes resulted in significant changes to the conversations between pair members. The shapes task elicited much shorter conversations containing more confidence-signaling statements and fewer stimulus-specific statements. There was a tendency in the CmC condition to use numbers to express confidence, perhaps to save time typing, or because nonverbal cues were unavailable. We conclude that when people are discussing things face-to-face, they are able to pick up on nonverbal confidence cues. When there is less opportunity to talk about features of the task—when comparing shapes, for example—pair members are more likely to mention their own confidence and to ask about their partners’ confidence. However, in the CmC condition, conversations tended to be very short, and confidence levels were not usually discussed explicitly, but coordination was achieved by the pair member with strong evidence typically suggesting an answer first.

It is remarkable that none of the individual difference variables assessed across Experiments 1 and 2 (assertiveness, need for cognition, need for closure, and dogmatism) were significantly related to persuasiveness or confidence. This suggests that persuasiveness was determined chiefly by our experimental manipulation of evidence strength, overwhelming all the personal characteristics that we investigated. However, in Experiment 3 we showed that participants with high trait dominance and low openness were more persuasive when they had weak evidence. This points to circumstances in which the confidence heuristic tends to break down and allow ignorance to overwhelm knowledge. However, this breakdown did not occur in the strong-evidence condition, where personality factors had no significant effect on persuasiveness. Overall, high confidence generated by strong evidence was more influential than any individual difference variable that we investigated in this study.

Experiment 3 showed, perhaps surprisingly, that the confidence heuristic operates similarly through both CmC and FtF. This suggests that confidence is communicated primarily through verbal signals and not through facial expressions, gestures, and tone of voice. However, verbal timing effects turned out to be significant: Our data showed that suggesting an answer first was strongly associated with persuasiveness, whether the speaker had strong or weak evidence, confirming findings of Gabbert et al. (2006) and Bang et al. (2014). Pair members with strong evidence were first to suggest an answer on almost three times as many trials as those with weak evidence. This response latency effect was also observed by Kimble and Seidel (1991), who found it to be associated with people’s confidence in answers to trivia questions, even in the absence of an audience. It suggests that people are able to recognize the strength of their own knowledge, and that they tend to avoid suggesting answers quickly when their knowledge is weak. Future research could examine whether starting the task at different times, with both members of a pair knowing that one of them has had longer to work on it, may disrupt the confidence heuristic.

The decision theorist Herbert Simon, who introduced the term *heuristic* in 1957, conceived it to be a rough-and-ready procedure or rule of thumb for making a decision or forming a judgment, without an exhaustive comparison of all available options, and hence without any guarantee of a correct or optimal result. Thomas and McFadyen (1995) presumably had this in mind when naming the confidence heuristic, and it does indeed seem to meet the definition, because it provides a rough-and-ready procedure for coordinating on a jointly desirable decision in the absence of sufficiently reliable information to guarantee certainty, especially for an individual with weak evidence, who nevertheless has a rule of thumb (follow the confidence cues) that leads to good decisions.

What are the implications of our investigation for future research? Our study raises many questions, some of which we have already mentioned. It is worth drawing attention to a few further unanswered research questions. First, we investigated common-interest games in which pair members were motivated to select the same targets from sets of stimuli. Whether the confidence heuristic operates in the same way when people have mixed motives—when they are motivated partly to compete and partly to cooperate with each other—is open for future researchers to discover. There will undoubtedly be situations in life where people hide their true confidence to gain a competitive advantage. Researchers studying this topic need to bear in mind the cooperative or competitive nature of the situation and whether the participants have reasons to trust or distrust each other.

Second, Experiment 1 revealed a possible trend that may also be worth investigating further. When the likely prediscussion difference in confidence between pair members, estimated from confidence ratings of the same materials observed in the pilot study, was relatively small (below about 25%), the pair member with strong evidence tended to be noticeably less persuasive than when the gap was larger. In future studies, it may be possible for researchers to establish a confidence threshold above which speakers tend to be persuasive, or a critical confidence gap between two people that enables the confidence heuristic to function effectively. Finally, researchers could examine cross-cultural differences in this area, as cultural norms around how confident one should appear, for example between Eastern and Western countries, could lead to cultural misunderstandings or lack of coordination and cooperation.

The confidence heuristic has important implications for everyday life, because it facilitates the emergence of truth in a cacophony of falsehood and misinformation. We agree with Price and Stone’s (2004) assertion that people tend to assume that “a more confident advisor makes more categorically correct judgments and is more knowledgeable” (p. 39). The finding that this works equally well through computer-mediated communication is important at a time when “fake news” is increasingly being disseminated face-to-face and through Facebook, Twitter, text messages, and other CmCs.

## Supplementary Material

10.1037/xge0000471.supp

## Figures and Tables

**Table 1 tbl1:** Percentages of Choices of Target and Nontarget Faces in the Pilot Study and Experiment 1 and Self-Rated Confidence in the Choices

	Pilot study				Experiment 1	
	Target		Nontarget		Target	
E-FIT #	Percentage choosing	*M* confidence	Percentage choosing	*M* confidence	Percentage choosing	*M* confidence
Weak-evidence E-FITS						
S9	n/a		100.00	39.97	35.71	53.82
M20	15.60	36.20	84.40	30.93	83.93	57.82
N2	6.30	10.00	93.70	38.21	87.50	58.75
L9	6.30	25.00	93.70	31.57	48.21	46.52
S8	15.60	35.00	84.40	41.40	42.86	65.21
M18	3.10	30.00	96.90	36.68	76.79	63.20
N7	n/a		100.00	35.56	67.86	54.30
L2	9.40	40.00	90.60	47.07	41.07	58.07
Average	7.04	29.37	92.96	37.67	60.49	57.21
Strong-evidence E-FITS						
S2	78.10	67.72	21.90	43.57	44.64	63.09
M5	87.50	82.21	12.50	52.50	92.86	81.16
N5	93.80	82.00	6.20	35.00	96.43	81.82
L5	65.60	58.81	34.40	37.27	50.00	56.89
S4	71.90	67.83	28.10	46.11	44.64	59.71
M3	71.90	78.17	28.10	48.56	83.93	78.64
N1	71.90	65.65	28.10	37.22	75.00	72.91
L4	46.90	62.33	53.10	50.00	41.07	63.27
Average	73.45	70.59	26.55	43.78	66.07	69.69
*Note.* n/a indicates mean confidence cannot be calculated as no-one chose the target.						

## Footnotes

1 Data, SPSS syntax files, and experimental materials are available from the OSF repository at https://osf.io/h4qfn/.

## A Formal Outline of the Confidence Heuristic

Thomas and McFadyen (1995) formalized the confidence heuristic as a two-player game in which the players choose between two alternatives, although the formalization generalizes naturally to multiplayer, multi-alternative decision problems. They began by assuming that two decision makers have common interests in coordinating their actions to achieve a jointly optimal outcome. Player 1 and Player 2 choose between two alternatives, A and B, and that their preferences coincide, so that the utilities associated with the alternatives—utilities being preferences as revealed or determined by the players’ choices—are *u*(A) and *u*(B) for both players. One of the alternatives has higher utility than the other, but the players do not know which is better. We assume two possible states of the world according to which alternative is better: α, in which *u*(A) > *u*(B), and β, in which *u*(B) > *u*(A).

Each player has some private evidence or information about the true state, and knows whether that evidence is relatively reliable or unreliable. Strong or reliable evidence that the state is α is denoted *s*_α_. Weak or unreliable evidence that the state is α is written *w*_α_; strong and weak evidence that the state is β are written *s*_β_ and *w*_β_ respectively. The probabilities of observing strong and weak evidence are π_*s*_ and π_*w*_ (with π_*s*_ + π_*w*_ = 1). For both players, we therefore have:

$$\begin{array}{l} \text{prob}\left(s_{\alpha }|\alpha \right)=\pi_{s}\times p,\text{prob}\left(s_{\beta }|\alpha \right)=\pi_{s}\times \left(1-p\right);\\ \text{prob}\left(w_{\alpha }|\alpha \right)=\pi_{w}\times q,\text{prob}\left(w_{\beta }|\alpha \right)=\pi_{w}\times \left(1-q\right);\\ \text{prob}\left(s_{\alpha }|\beta \right)=\pi_{s}\times \left(1-p\right),\text{prob}\left(s_{\beta }|\beta \right)=\pi_{s}\times p;\\ \text{prob}\left(w_{\alpha }|\beta \right)=\pi_{w}\times \left(1-q\right),\text{prob}\left(w_{\beta }|\beta \right)=\pi_{w}\times q; \end{array}$$

where 1 > *p* > *q* > 1/2, by definition of strong and weak evidence.

Both players assign a prior probability of 1/2 to α and β and then used Bayes’ rule to update their subjective probabilities after considering their private evidence. For example, after receiving private evidence *s*_α_, a player’s posterior belief becomes

$$\text{prob}\left(\alpha |s_{\alpha }\right)=\frac{\text{prob}\left(\alpha {\text{ and}\text{ s}}_{\alpha }\right)}{\text{prob}\left(s_{\alpha }\right)}=\frac{\left(\pi_{s}\times p\right)/2}{\left(\pi_{s}\times p\right)/2+\pi_{s}\left(1-p\right)/2}=p.$$

After receiving strong evidence *s*_α_, a player’s probability of α increases from 1/2 to *p* > 1/2, and after receiving weak evidence *w*_α_, the probability goes from 1/2 to *q* > 1/2 (with *q* < *p*). Thus, we have

$$\begin{array}{l} \text{prob}\left(\alpha |s_{\alpha }\right)=\text{prob}\left(\beta |s_{\beta }\right)=p,\\ \text{prob}\left(\alpha |w_{\alpha }\right)=\text{prob}\left(\beta |s_{\beta }\right)=q. \end{array}$$

Using *b*_*i*_ to represent the posterior belief of Player *i* about the state α, we have *b*_*i*_ = *p* after observing *s*_α_, *b*_*i*_ = (1 – *p*) after observing *s*_β_, *b*_*i*_ = *q* after observing *w*_α_, *b*_*i*_ = (1 – *q*) after observing *w*_β_. With these posterior beliefs, the two players meet and discuss the problem, and then each player decides separately which alternative to choose. If they were able to combine their private information, then a simple application of Bayes’ rule yields

$$d_{1}=d_{2}={\left(1+\frac{1-b_{1}}{b_{1}}\times \frac{1-b_{2}}{b_{2}}\right)}^{-1},$$ 1

where *d*_*i*_ is Player *i*’s postdiscussion belief (subjective probability) regarding the likelihood of α. If their information could be combined or pooled without error, then both players converge on identical beliefs. A simple decision rule that maximizes the expected utility of both players is for each to choose A if *d*_*i*_ > 1/2, B if *d*_*i*_ < 1/2, and either alternative if *d*_*i*_ = 1/2. If their posterior beliefs cause them to prefer different alternatives before discussing the problem with each other, and if one has stronger evidence than the other, then this decision rule will result in both choosing the alternative associated with the stronger evidence. Thus, if Player 1 observes *s*_α_ and Player 2 observes *w*_β_, then *b*_1_ = *p*, *b*_2_ = 1 – *q*, and from Equation 1 we have *d*_*i*_ > 1/2, and both players choose A.

How do the players implement this jointly optimal decision? Thomas and McFadyen (1995) assume that, when they discuss the problem with each other, they can communicate their prior beliefs (on the basis of their weak or strong evidence) in either a tentative (T) or confident (C) manner. A communication strategy is a choice of T_*k*_ or C_*k*_ when communicating to the other players about alternative *k.* In the simple binary-choice example that we have been examining, *k* ∈ {A, B}. After communicating with each other, the players separately choose one of the alternatives A or B, using a decision function *D* based on the players’ communication of their confidence.

A natural decision rule, using functional notation, is to choose the alternative that has been most confidently communicated. This implies the following:

$$D\left(T_{A},T_{A}\right)=D\left(C_{A},C_{A}\right)=D\left(C_{A},T_{B}\right)=D\left(T_{B},C_{A}\right)=A,$$ 2

$$D\left(T_{B},T_{B}\right)=D\left(C_{B},C_{B}\right)=D\left(C_{B},T_{A}\right)=D\left(T_{A},C_{B}\right)=B\text{.}$$ 3

Here “*D*(*x*, *y*) = *z*” should be read as a decision function *D* that assigns either A or B to every pair of tentative or confident communication styles of the two players. If the alternatives are communicated with equal confidence, *D*(*T*_A_, *T*_B_) or *D*(*C*_A_, *C*_B_), then the decision rule specifies that each player chooses between the alternatives randomly, and in that case the rule does not yield a determinate solution. Equations 2 and 3 define the confidence heuristic and result in players converging on the alternatives that maximize their individual expected utilities.
