# Supplementary material for: The Persuasive Power of Knowledge: Testing the Confidence Heuristic
Source: J Exp Psychol Gen. 2018 Aug 23;147(10):1431–44. doi: 10.1037/xge0000471 (PMC6166527; doi:10.1037/xge0000471)
Supplement: Supplementary file 4 [file ConfidenceSuppMaterials.zip › ConfidenceSuppMaterials.doc]

**SUPPLEMENTARY MATERIALS FOR:**

**The Persuasive Power of Knowledge: Testing the Confidence Heuristic**

##### EXPERIMENT 1 ANSWER SHEET

#### Pno_______

#### My Age:__________

#### My Gender: MALE / FEMALE Gender of partner: MALE / FEMALE

#### My Folder: GREEN / BLUE

Relationship with your partner in this experiment (circle one):

3: Regular acquaintance	4: Friend

2: Weak acquaintance	5: Good friend

1: Complete strangers	6: Intimate partner

**THE POLICE AND SUSPECTS PROBLEM**

##### Imagine that you and your partner in the experiment are two police officers. A crime has been committed and two witnesses have just been interviewed - one interviewed by you and the other interviewed by the other police officer. Each witness has created an e-fit picture of the criminal.

##### You must decide who to arrest for the crime. There are nine suspects shown on the grid of faces. You may exchange verbal information but not see each other’s e-fits.

##### Eyewitnesses can often differ from each other because they have different views of the event and the criminal, so the e-fits created may be good or not-so-good likenesses of the suspect.

**Payment:**

- If you both choose the same face, of the person who did commit the crime, you will get **40p each.**
- If you both choose the same but innocent person then you get **20p each**.
- If you choose two different people, then you **each get nothing**, even if one of you chooses the person who is guilty.

##### You must choose a suspect’s face - you cannot leave a blank.

##### There are 23 e-fits to look at and 4 grids of 9 suspects’ faces to examine. You will be given an example grid and e-fit to start off with to ensure you understand the procedure.

##### You have UP TO 2 minutes to discuss each e-fit with your partner, but no longer. You can take less time if you want to.

Pno_______

For the set of faces, please look at the e-fit and decide which of the faces on the grid you think it looks MOST like. Discuss with your partner your preliminary opinion, then, before the 2 minute time limit is up:

1) Write down the number of the person that you think it is from 1-9 next to the letter A, B, C… below to match the one that appears on the bottom right hand corner of your e-fit.
2) Write any number from 0-100 to show how confident you feel that you chose the correct person from the lineup.

0-----------------------------------50--------------------------------------100
not at all sure	completely sure
that I chose the	that I chose the
right person	right person.

| E-fit | **Person** | Confidence |  | E-fit | **Person** | Confidence |  |
| --- | --- | --- | --- | --- | --- | --- | --- |
| Example |  |  |  |  | | |  |
| GRID **S** | | |  | GRID **M** | | |  |
| A |  |  |  | Q |  |  |  |
| B |  |  |  | R |  |  |  |
| C |  |  |  | S |  |  |  |
| GRID **M** | | |  | GRID **N** | | |  |
| D |  |  |  | W |  |  |  |
| E |  |  |  | X |  |  |  |
| F |  |  |  | Y |  |  |  |
| GRID **N** | | |  | GRID **L** | | |  |
| I |  |  |  | Z |  |  |  |
| J |  |  |  | AA |  |  |  |
| GRID **L** | | |  | BB |  |  |  |
| K |  |  |  |  | | |  |
| L |  |  |  |  |  |  |  |
| M |  |  |  |  |  |  |  |
| GRID **S** | | |  |  | | | |
| N |  |  |  | You have now completed this task. Thank you.  Now please complete the next questionnaire while your scores and cash are calculated. | | | |
| O |  |  |  |  |  |  |  |
| P |  |  |  |  |  |  |  |
|  |  |  |  |  |  |  |  |

##### EXPERIMENTS 2 & 3 ANSWER SHEET

Pno_______

Tested by______________

#### My Age:__________

#### My Gender: MALE / FEMALE Gender of partner: MALE / FEMALE

#### My Folder: A / B Partner’s Pno:__________

Relationship with your partner in this experiment (**circle one**):

**3**: Regular acquaintance	**4**: Friend

**2**: Weak acquaintance	**5**: Good friend

**1**: Complete strangers	**6**: Intimate partner

**Size Discussion Task**

##### You are seated with a partner, and after a discussion with them you must take a decision about which answer you believe to be correct for a series of shapes. You can earn cash for each of your answers, but how much you each get will depend not only on your own answer but also on the answers given by both of you.

PLEASE READ THIS CAREFULLY:

**Payment:**

- If you both choose the **same answer and it is correct**, you will get **20p each.**
- If you both choose the **same answer but it is incorrect** then you get **10p each**.
- If you choose a **different answer** to the other person, then you **each get nothing**, even if one of you chooses the correct answer.

##### You must choose an answer - you cannot leave a blank.

##### You have UP TO 2 minutes to discuss each task with your partner, but no longer. You can take less time if you want to.

Pno_______

##### SHAPES

For the next 16 questions your task is to look at the shapes A and B and decide which of them you think is **closest in size to the shape on the far right** of them. Before you write down your answer, discuss with your partner your preliminary opinion, then, **before the 2 minute time limit is up**:

1) Circle your chosen answer.

2) Write any number from 0-100 to show how confident you feel that you chose the correct answer, using the following scale:

0-----------------------------------50--------------------------------------100
not at all sure	completely sure
that I chose the	that I chose the
right answer	right answer.

|  | SHAPES | | |  | SHAPES | | |
| --- | --- | --- | --- | --- | --- | --- | --- |
|  | **Task** | **answer** | Conf |  | **Task** | **answer** | Conf |
|  | 1 | A / B |  |  | 9 | A / B |  |
|  | 2 | A / B |  |  | 10 | A / B |  |
|  | 3 | A / B |  |  | 11 | A / B |  |
|  | 4 | A / B |  |  | 12 | A / B |  |
|  | 5 | A / B |  |  | 13 | A / B |  |
|  | 6 | A / B |  |  | 14 | A / B |  |
|  | 7 | A / B |  |  | 15 | A / B |  |
|  | 8 | A / B |  |  | 16 | A / B |  |

You have now completed this task. Thank you.

Now please complete the next questionnaire while your scores and cash are calculated.
